# Impact of COVID‐19 pandemic on the management of nonculprit lesions in patients presenting with ST‐elevation myocardial infarction: Outcomes from the pan‐London heart attack centers

**DOI:** 10.1002/ccd.30056

**Published:** 2021-12-30

**Authors:** Ozan M. Demir, Callum D. Little, Richard Jabbour, Haseeb Rahman, Max Sayers, Asrar Ahmed, Michelle J. Connolly, Ritesh Kanyal, Philip MacCarthy, Simon J. Wilson, Miles Dalby, Ajay Jain, Iqbal Malik, Roby Rakhit, Divaka Perera

**Affiliations:** ^1^ NIHR Biomedical Research Centre and British Heart Foundation Centre of Excellence, School of Cardiovascular Medicine and Sciences King's College London London UK; ^2^ Department of Cardiology Royal Free London NHS Foundation Trust London UK; ^3^ Department of Cardiology Imperial College Healthcare NHS Foundation Trust London UK; ^4^ Department of Cardiology Barts Health NHS Trust London UK; ^5^ Department of Cardiology Royal Brompton and Harefield NHS Foundation Trust London UK; ^6^ Department of Cardiology St George's University Hospitals NHS Foundation Trust London UK; ^7^ Department of Cardiology King's College Hospital NHS Foundation Trust London UK

**Keywords:** bystander, coronary physiology, COVID‐19, FFR, nonculprit, STEMI

## Abstract

**Background:**

The impact of COVID‐19 on the diagnosis and management of nonculprit lesions remains unclear.

**Objectives:**

This study sought to evaluate the management and outcomes of patients with nonculprit lesions during the COVID‐19 pandemic.

**Methods:**

We conducted a retrospective observational analysis of consecutive primary percutaneous coronary intervention (PPCI) pathway activations across the heart attack center network in London, UK. Data from the study period in 2020 were compared with prepandemic data in 2019. The primary outcome was the rate of nonculprit lesion percutaneous coronary intervention (PCI) and secondary outcomes included major adverse cardiovascular events.

**Results:**

A total of 788 patients undergoing PPCI were identified, 209 (60%) in 2020 cohort and 263 (60%) in 2019 cohort had nonculprit lesions (*p* = .89). There was less functional assessment of the significance of nonculprit lesions in the 2020 cohort compared to 2019 cohort; in 8% 2020 cohort versus 15% 2019 cohort (*p* = .01). There was no difference in rates of PCI for nonculprit disease in the 2019 and 2020 cohorts (31% vs 30%, *p* = .11). Patients in 2020 cohort underwent nonculprit lesion PCI sooner than the 2019 cohort (*p* < .001). At 6 months there was higher rates of unplanned revascularization (4% vs. 2%, *p* = .05) and repeat myocardial infarction (4% vs. 1%, *p* = .02) in the 2019 cohort compared to 2020 cohort.

**Conclusion:**

Changes to clinical practice during the COVID‐19 pandemic were associated with reduced rates of unplanned revascularization and myocardial infarction at 6‐months follow‐up, and despite the pandemic, there was no difference in mortality, suggesting that it is not only safe but maybe more efficacious.

## 1 INTRODUCTION

The COVID‐19 pandemic necessitated an unprecedented restructuring of clinical pathways in cardiac centers globally. In the UK, there was a significant reduction in elective admissions in an effort to create capacity lacking for COVID‐19 patients. For interventional cardiology, this culminated in reduced capacity for elective procedures.^1^ Concomitantly, patients with COVID‐19 presenting with ST‐elevation myocardial infarction (STEMI) were shown to have increased incidence and burden of thrombotic culprit lesions.^2^ However, it is not known whether COVID‐19 has impacted the diagnosis and management of nonculprit lesions. Of note, randomized trials involving more than 6300 patients over the last decade all agree that revascularization of noculprit lesions at STEMI is superior to medical therapy alone.^3^ However, the role of coronary physiology in this setting for the assessment of nonculprit lesions remains unclear. Recently, the FLOWER‐MI study attempted to answer this question by utilization of coronary physiology to adjudicate stenosis severity in the setting of STEMI. It showed that there was no difference in the primary endpoint which was a composite of all‐cause death, nonfatal myocardial infarction, or unplanned hospitalization leading to urgent revascularization at 1 year between fractional flow reserve (FFR)‐ and angiography‐based management.^4^ We hypothesized that the rate of interventional treatment of nonculprit lesions during the pandemic would be lower than it was beforehand, and that rate of coronary physiology utilization would decrease.

## 2 METHODS

### 2.1 Study design and patient population

We conducted a retrospective observational analysis of consecutive primary percutaneous coronary intervention (PPCI) pathway activations to all seven heart attack centers in London, UK. The PPCI programme in London is the largest urban network of seven heart attack centres in the UK using a single ambulance triggered service and providing 24/7 treatment for STEMI to a population of 9 million. The study period was March 1st to April 30th, 2020, corresponding with the peak of daily reported COVID‐19 cases in the UK. A control period of March 1st to April 30th, 2019 was used for comparison. Of patients presenting via the PPCI pathway during the study period, we included those with (1) an electrocardiographic consistent with STEMI^5^; and (2) a culprit infarct‐related lesion on coronary angiography requiring intervention. Patients who did not present via the PPCI pathway, such as those self‐presenting to the hospital or those developing STEMI as an inpatient were not included. Patients who underwent coronary angiography revealing unobstructed coronary vessels and/or those who were given an alternative diagnosis were excluded. Data were collected from the local British Cardiac Intervention Society (BCIS) databases. Patients were included if they underwent PPCI for a STEMI and were found to have further disease in a noninfarct related artery.^2^

### 2.2 BCIS—National Institute for Cardiovascular Outcomes Research Database

The BCIS—National Institute for Cardiovascular Outcomes Research Database collects data from all hospitals performing percutaneous coronary intervention (PCI) in UK.^6^ Data are collected prospectively at each hospital, electronically encrypted, and transferred online to a central database. Patients' survival data are obtained by linkage of patients' National Health Service numbers to the Office of National Statistics.

### 2.3 Outcomes

The primary outcome was the rate of nonculprit lesion PCI and secondary outcomes included major adverse cardiovascular events (at 30 days and 6 months) and procedural timing and characteristics. Major adverse cardiovascular events and all‐cause mortality during STEMI‐related hospitalization were determined from electronic patient records and discharge summaries. In addition, baseline demographic characteristics were also retrieved. All events are reported cumulatively at respective time points.

### 2.4 Statistical analysis

Normality of data was assessed by the histogram, normal Q–Q plot, and Shapiro–Wilk test. Continuous normal data are expressed as mean ± standard deviation and compared using paired Student's *t* tests. Nonnormal data are expressed as median (interquartile range) and compared using the Mann–Whitney test. Categorical data were presented as numbers with percentages and compared using the *χ* ^2^ test. A *p* < 0.05 was deemed to be of statistical significance. Data analysis was performed using SPSS 27 (IBM Corp).

## 3 RESULTS

A total of 788 patients undergoing primary PCI were identified, 348 during 2020 and 440 during 2019 study periods. Of these, 209 (60%) in 2020 cohort and 263 (60%) in 2019 cohort had nonculprit lesions (*p* = .89). No differences between 2019 and 2020 cohorts were identified in terms of baseline characteristics or the delays from onset of chest pain to the first call for help or door‐to‐balloon time (Table 1). However, first‐call‐to‐door time was significantly longer in the 2020 cohort compared with the 2019 cohort (*p* = .001).

**Table 1:** Baseline characteristics

	2020 Cohort	2019 Cohort	*p*
	(*n* = 209)	(*n* = 263)	
Baseline characteristics			
Age (years)	64 (57–72)	64 (57–75)	0.46
Male sex	168 (80%)	207 (79%)	0.74
Diabetes mellitus	55 (26%)	70 (27%)	0.94
Hypertension	120 (57%)	127 (48%)	0.05
Hyperlipidemia	78 (37%)	107 (41%)	0.46
Smoking history	88 (42%)	155 (59%)	<0.001
Previous myocardial infarction	31 (15%)	40 (15%)	0.91
Stroke	7 (3%)	12 (5%)	0.51
Previous PCI	30 (15%)	42 (16%)	0.63
Previous CABG	1 (1%)	7 (3%)	0.07
Peripheral vascular disease	2 (2%)	4 (3%)	0.61
Renal disease	8 (4%)	9 (3%)	0.81
Family history of IHD	27 (13%)	39 (15%)	0.55
COVID‐19 positive	31 (15%)	–	–
PPCI pathway characteristics			
Chest pain to first call time (min)	80 (30–352)	90 (23–242)	0.81
The first callto door time (min)	87 (65–121)	76 (60–96)	0.001
Door to balloon time (min)	47 (34–65)	49 (35–70)	0.24
Total ischemic time (min)	307 (172–589)	252 (163–548)	0.18
Out of hospital cardiac arrest	20 (10%)	18 (7%)	0.28
Cardiogenic shock	24 (12%)	32 (12%)	0.83

Abbreviations: CABG, coronary artery bypass grafting; IHD, **ischemic heart disease; PCI, percutaneous coronary intervention; PPCI, primary percutaneous coronary intervention.

**Figure 1:**
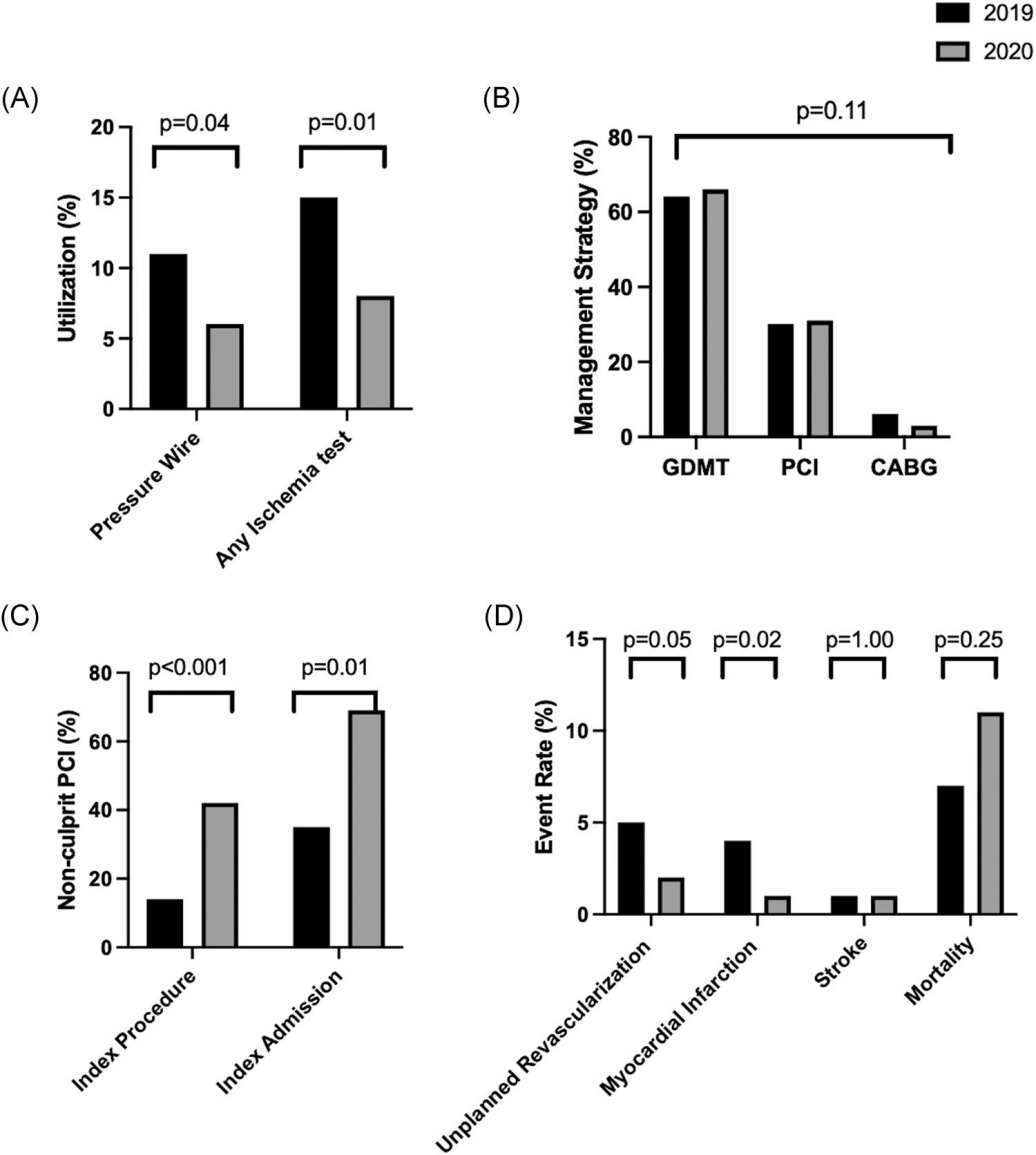
Management and outcome of nonculprit lesions in 2019 and 2020. (A) Utilization of ischaemia testing before revascularization; (B) management strategy; (C) nonculprit PCI at index procedure and index admission; and (D) 6‐months outcomes. CABG, coronary artery bypass grafting; GDMT, guideline‐directed medical therapy; PCI, percutaneous coronary intervention

Nonculprit lesion characteristics were similar, including number, location, and severity of the lesion(s) (Table 2). There was the less functional assessment of the significance of nonculprit lesions in the 2020 cohort compared to the 2019 cohort; a pressure wire or a noninvasive ischemia test was utilized in 8% 2020 cohort versus 15% 2019 cohort (*p* = .01) (Table 1). There was no difference in rates of PCI for nonculprit disease in the 2019 and 2020 cohorts (31% vs. 30%, *p* = .11). Patients in 2020 cohort underwent nonculprit lesion PCI sooner than the 2019 cohort, 2 (0–49) versus 54 (5–103) days (*p* < .001). In addition, more patients in 2020 underwent nonculprit lesion PCI during the index procedure itself (27 [42%] vs. 11 [14%], *p* < .001) and during the index admission (45 [69%] vs. 27 {35%], *p* = .01) than in 2019 (Table 3). There was no difference in the number of vessels, number of lesions, number of stents, length of the stent, or largest balloon used between cohorts. The rate of major adverse cardiac events was similar at 30 days in the two cohorts, 11% versus 12% (*p* = .42), 2020 versus 2019, respectively. However, at 6 months, there were higher rates of unplanned revascularization and repeat myocardial infarction in the 2019 cohort compared to the 2020 cohort (Figure 1). In‐hospital 30‐day and 6‐month mortality were similar in both cohorts; of the 20 patients, in the 2020 cohort who died during the index admission, 8 had positive COVID‐19 tests at the time.

**Table 2:** Characteristics of nonculprit lesions

	2020 Cohort	2019 Cohort	*p*
	(*n* = 209)	(*n* = 263)	
Nonculprit lesion characteristics			
Number of nonculprit lesions	1.51 ± 0.62	1.60 ± 0.65	0.16
Nonculprit lesions(s)			
1‐vessel	116 (55%)	127 (48%)	0.28
2‐vessel	79 (38%)	113 (43%)	
3‐vessel	14 (7%)	23 (9%)	
Nonculprit vessel			
Left main stem	22 (7%)	25 (6%)	0.60
Left anterior descending	112 (35%)	144 (34%)	
Left circumflex	94 (30%)	131 (31%)	
Intermediate	4 (1%)	7 (2%)	
Right coronary	84 (27%)	115 (27%)	
Nonculprit lesion severity			
<50%	81 (26%)	84 (20%)	0.49
≥50%–70%	73 (23%)	111 (26%)	
≥70%–90%	79 (25%)	121 (29%)	
>90%	76 (24%)	87 (21%)	
100% (CTO)	7 (2%)	19 (4%)	
Nonculprit lesion ischemia test			
Pressure wire utilization			
Per patient	12 (6%)	29 (11%)	0.04
Per vessel	15 (5%)	36 (9%)	0.04
Pressure wire or noninvasive ischemia test	16 (8%)	40 (15%)	0.01

Abbreviation: CTO, chronic total occlusion.

**Table 3:** Management of nonculprit lesions and outcomes

	2020 Cohort	2019 Cohort	*p*
	(*n* = 209)	(*n* = 263)	
Management of nonculprit lesion			
Management (per patient)			
GDMT	139 (66%)	169 (64%)	0.11
PCI	65 (31%)	77 (30%)	
CABG	5 (3%)	17 (6%)	
Time to nonculprit lesion PCI (days)	2 (0–49)	54 (5–103)	<0.001
Number of vessel(s) treated			
1‐vessel	56 (92%)	64 (90%)	0.47
2‐vessel	4 (7%)	6 (9%)	
3‐vessel	1 (1%)	1 (1%)	
Number of lesions treated	1.36 ± 0.66	1.31 ± 0.62	0.65
Number of stents implanted	1.46 ± 0.87	1.45 ± 0.89	0.96
Stented segment (mm)	28.8 ± 10.3	32.0 ± 15.3	0.18
Largest balloon/stent (mm)	3.7 ± 2.0	3.2 ± 0.67	0.11
Outcomes			
In‐hospital mortality	20 (10%)	16 (6%)	0.67
Length of stay	3 (2–4)	3 (2–5)	0.03
30 days			
Unplanned revascularization	3 (1%)	10 (4%)	0.12
Myocardial infarction	0 (0%)	5 (2%)	0.07
Stroke	0 (0%)	1 (1%)	0.26
Mortality	21 (10%)	15 (6%)	0.08
6 months			
Unplanned revascularization	4 (2%)	14 (5%)	0.05
Myocardial infarction	1 (1%)	10 (4%)	0.02
Stroke	1 (1%)	2 (1%)	1.00
Mortality	22 (11%)	19 (7%)	0.25

Abbreviations: CABG, coronary artery bypass grafting; GDMT, guideline‐directed medical therapy; PCI, percutaneous coronary intervention.

## 4 DISCUSSION

The main findings from our study are that during the COVID‐19 pandemic (2020 cohort): a) there was no difference in the rate of nonculprit lesion PCI; b) the timing of nonculprit PCI was more frequently performed during the index procedure; c) there was less utilization of functional testing to assess the significance of nonculprit lesions; d) there was a reduction in the length of stay; and e) there were lower rates of unplanned revascularization and repeat myocardial infarction at 6 months.

Our results demonstrate that the rate of nonculprit lesion PCI remained at approximately 30% despite the onset of the pandemic. However, there was significantly greater nonculprit PCI performed at the index procedure and/or the index admission during the COVID‐19 pandemic. This increased performance of nonculprit lesion PCI was likely driven by the desire to limit the amount of time spent by patients in the hospital and the limitations to elective outpatient PCI during the peak of the COVID‐19 pandemic. Of note, a substantial body of published observational and randomized controlled data have demonstrated that “preventative PCI” of nonculprit lesions is associated with better outcomes, compared with culprit vessel‐only PCI in patients with STEMI, primarily driven by a lower risk of reinfarction.^7^ The CvLPRIT study demonstrated that nonculprit lesion revascularization at index admission was associated with a reduction in overall adverse clinical events compared to culprit‐only revascularization.^8^ Recently, the COMPLETE trial demonstrated that complete revascularization was superior to culprit‐lesion‐only PCI in reducing the risk of cardiovascular death or myocardial infarction, as well as ischemia‐driven revascularization. Furthermore, the benefit of complete revascularization was consistently observed regardless of the timing of nonculprit‐lesion PCI, index hospitalization, or after hospital discharge.^9^ Meta‐analysis of 10 randomized clinical trials of 7030 unique patients demonstrated a significant reduction, 31% relative reduction in cardiovascular mortality.^10^ This is supported by current European Society of Cardiology and American Heart Association/American College of Cardiology guidelines, providing a Class IIa recommendation for nonculprit lesion PCI at index admission.^11^, ^12^ Our study builds on these studies by providing a real‐world preventative PCI cohort that was necessitated by the global COVID‐19 pandemic which is supportive of these findings. The uncertainty regarding the availability of elective outpatient PCI during the peak of the COVID‐19 pandemic is likely to have had a significant clinical impact resulting in changes to a routine practice whereby median time to nonculprit lesion PCI was reduced significantly from a median of 54 days to 2 days.

The COVID‐19 pandemic could have resulted in multiple delays in PPCI pathways. Our analysis comparing the 2020 study cohort and the 2019 control cohort importantly revealed comparable door‐to‐balloon times between the two groups but prolonged first‐call‐to‐door time in the 2020 cohort. Suggesting that modifications to the existing in‐hospital PPCI pathways, such as routine use of personal protective equipment (PPE) neither delayed the time taken to achieve coronary revascularization (door‐to‐balloon time) nor resulted in worse outcomes. It is plausible that the increased first‐call‐to‐door time allowed clinical teams greater time to don full PPE, this was standard practice for all cardiac catheterization laboratory staff.^13^ In our study, all heart attack centers were supported by the London Ambulance Service, which has protocolized admissions pathway, resulting in cardiac catheterization laboratory staff being informed of patients on‐route with potential acute coronary syndromes. Hence, resulting in no difference in door‐to‐balloon time during the COVID‐19 pandemic despite the need for PPE.

The COVID‐19 pandemic resulted in a significant reduction in pressure wire‐based physiological assessment of lesion severity. However, compared to previously published data, the 2019 cohort had lower rates of coronary physiology assessment.^14^, ^15^ This may be attributable to the link between functional stenosis assessment and revascularization in STEMI setting remains unclear. The recently published FLOWER‐MI, an investigator‐initiated, multicentre trial conducted over 2 years in France, randomized 1171 patients following STEMI with angiographic stenosis of ≥50% in at least one nonculprit artery to FFR or angiographic guided PCI. All patients underwent nonculprit assessment before discharge. There was no difference in the primary endpoint which was a composite of all‐cause death, nonfatal myocardial infarction, or unplanned hospitalisation leading to urgent revascularization at 1 year (hazard ratio, 1.32 for FFR vs. angiography; 95% confidence interval, 0.78–2.23).^4^ As our data represents real‐world practice it does not elucidate this point except potentially to indicate that reduction in coronary physiology utilization was not associated with worse clinical outcomes. However, this may have been confounded by outcome benefits attributable to concomitant early preventative PCI.

Our study presents some limitations. First, its retrospective design nature makes it susceptible to the usual types of bias ascribed to this design. Second, we only included patients receiving PCI for STEMI, and data on those subjects with STEMI who did not undergo PCI (including those receiving systemic thrombolysis only and those managed medically in the first instance) were not included in our analysis. Third, the threshold used by emergency service to initiate the PPCI pathway, during the pandemic, may have been influenced by factors relating to infection control and resource management. This may have contributed to the reduction seen in PPCI activations worldwide in 2020. Fourth, its observational design includes potential selection bias due to a reduction in STEMI admission in 2020 compared to 2019, whereby higher‐risk patients potentially did not make it to the hospital. Fifth, the relatively small size of the two cohorts with nonculprit lesions may have impacted findings in this study. Finally, the outcome of those patients who did not present to the PPCI service is unknown. They may have significantly worse late outcomes, with heart failure, arrhythmia, and death as yet unmeasured in the community.

## 5 CONCLUSION

In our study, changes to clinical practice during the COVID‐19 pandemic were associated with reduced rates of unplanned revascularization and myocardial infarction at 6‐months follow‐up, and despite the pandemic, there was no difference in mortality, suggesting that it is not only safe but may be more efficacious. This could also have significant resource implications even beyond the pandemic.

## CONFLICT OF INTERESTS

The authors declare that there are no conflict of interests.
